# Stress and viral insults do not trigger E200K PrP conversion in human cerebral organoids

**DOI:** 10.1371/journal.pone.0277051

**Published:** 2022-10-27

**Authors:** Anna Smith, Bradley R. Groveman, Clayton Winkler, Katie Williams, Ryan Walters, Jue Yuan, Wenquan Zou, Karin Peterson, Simote T. Foliaki, Cathryn L. Haigh

**Affiliations:** 1 Laboratory of Persistent Viral Diseases, Division of Intramural Research, National Institute of Allergy and Infectious Diseases, Rocky Mountain Laboratories, National Institutes of Health, Hamilton, MT, United States of America; 2 Department of Pathology, Case Western Reserve University School of Medicine, Cleveland, OH, United States of America; University of Minnesota Medical Center: University of Minnesota Health, UNITED STATES

## Abstract

Prion diseases are a group of rare, transmissible, and invariably fatal neurodegenerative diseases that affect both humans and animals. The cause of these diseases is misfolding of the prion protein into pathological isoforms called prions. Of all human prion diseases, 10–15% of cases are genetic and the E200K mutation, which causes familial Creutzfeldt-Jakob disease (CJD), is the most prevalent. For both sporadic and genetic disease, it remains uncertain as to how initial protein misfolding is triggered. Prior studies have linked protein misfolding with oxidative stress insults, deregulated interactions with cellular cofactors, and viral infections. Our previous work developed a cerebral organoid (CO) model using human induced pluripotent stem cells containing the E200K mutation. COs are three-dimensional human neural tissues that permit the study of host genetics and environmental factors that contribute to disease onset. Isogenically matched COs with and without the E200K mutation were used to investigate the propensity of E200K PrP to misfold following cellular insults associated with oxidative stress. Since viral infections have also been associated with oxidative stress and neurodegenerative diseases, we additionally investigated the influence of Herpes Simplex Type-1 virus (HSV1), a neurotropic virus that establishes life-long latent infection in its host, on E200K PrP misfolding. While COs proved to be highly infectable with HSV1, neither acute nor latent infection, or direct oxidative stress insult, resulted in evidence of E200K prion misfolding. We conclude that misfolding into seeding-active PrP species is not readily induced by oxidative stress or HSV1 in our organoid system.

## Introduction

Prion diseases are caused by the misfolding of the native prion protein (PrP^C^) into the pathogenic and infectious isoforms PrP^Sc^, which oligomerize resulting in protein aggregation and vacuole formation in the brain. Disease can present as dementia, loss of motor control and psychiatric symptoms before progressing into a coma and death [1,2]. Human prion diseases include Kuru, Gerstmann-Sträussler-Scheinker syndrome (GSS), fatal familial insomnia (FFI), and Creutzfeldt-Jakob disease (CJD) [3]. Of these CJD is the most common, affecting approximately 1 person per million worldwide [1]. Genetic prion diseases account for about 10–15% of cases. The majority of these are associated with a glutamic acid to lysine mutation at codon 200 (E200K) in the PrP^C^ gene (*PRNP*), which has a penetrance of up to 100% [2,4]. Despite the knowledge that *PRNP* E200K carriers have a very high likelihood of developing disease, the events that trigger the start of protein misfolding are unknown. The age of onset of disease is also highly variable, indicating that other genetic or environmental factors might influence the initiating events.

When considering what triggers PrP^C^ misfolding and oligomerization, isolated experiments have implicated oxidative stress as well as dysfunctional binding of different cofactors including nucleic acids, glycosaminoglycans, and redox metals [5–9]. One hypothesis is that these perturbations may destabilize the protein enough to induce conversion [9]. Mutations within PrP have been shown to destabilize its normal folded structure [10,11], potentially rendering it more susceptible to unfolding and refolding incorrectly. Oxidation of methionine residues within E200K PrP amino acid sequence resulting from oxidative stress is associated with destabilization of PrP and occurs sequentially from methionine 213 until the inner hydrophobic core is disrupted [12,13]. Furthermore, a study by Zhang and colleagues found that PrP with the E200K mutation shows significantly altered electrostatic surface potential, which may contribute to dysregulated interactions with cofactors and other molecules [14]. Therefore, changed cellular redox balance or cofactor availability for PrP binding could be early events in prion misfolding.

Various intrinsically produced oxidative stress by-products are recognized to play a role in neurodegenerative diseases. Four-Hydroxynonenal (HNE), one of the most prevalent byproducts of lipid peroxidation, is considered a marker of oxidative stress and has been implicated in tissue damage, aging, cellular dysfunction, inflammation and other neurodegenerative diseases including Alzheimer’s and Parkinson’s diseases [15]. A study done by Andreoletti and colleagues found that HNE accumulated primarily in the astrocytes of prion infected mice and caused protein adducts throughout the brain [16]. Methylglyoxal is another highly reactive by-product generated during glycolysis and is produced abundantly in the brain, due to the brain’s high energy expenditure. It has also been observed at increased levels in conditions of oxidative stress [17–20]. Similar to HNE, methylglyoxal has been linked to diabetes, cancer, inflammation, and aging, and is thought to cause cell damage and produce free radicals, which contribute to oxidative stress [20]. Byproducts of oxidative metabolism, such as the aforementioned stressors, could have the potential to destabilize E200K PrP.

There are numerous situations in the brain that might result in a burst of reactive oxygen species (ROS), such as ischemia and viral infections. As well as oxidative stress, viral infections are known to induce inflammation, and to dysregulate vital cell processes. Viruses also interact with endogenous (and introduce foreign) cellular co-factors such as nuclei acids and glycosaminoglycans [21]. Herpes Simplex Virus Type-1 (HSV-1) is highly infectious and is known to establish life-long latent infection in neurons following acute infection [22]. It is estimated to infect more than two thirds of the global population, and in some countries, more than 90% [22]. Studies have shown that active infection causes oxidative stress, damages DNA, impairs DNA repair, disrupts proapoptotic and mitochondrial biogenesis signaling, stimulates amyloid plaque deposition, induces neuroinflammation and neurotoxicity, and introduces foreign molecules which may interact negatively with PrP^C^ [21]. Latent infection is thought to cause oxidative damage and neuroinflammation, and chronic viral reactivations from stressful triggers can result in cumulative damage [23,24]. Meta analysis has further linked HSV-1 to the development of Alzheimer’s and other neurodegenerative diseases [21]. With regard to prion diseases, intrathecally synthesized HSV antibodies were found in 3 of 25 CJD patients analysed [25]. Two case studies also found CJD with concurrent HSV infection [26]. While none of these data suggest HSV causes prion disease, its role in prion pathogenesis has not yet been investigated in this context.

Cerebral organoids (COs) are spheres of neuronal lineage tissue that can be differentiated from human induced pluripotent stem cells (iPSCs). COs progressively mature with neuronal populations differentiating first, then astrocytes and oligodendrocytes appearing from approximately 2–5 months old [27]. Our group has previously shown that COs can take up and propagate sporadic CJD, but do not spontaneously generate genetic prion disease despite demonstrating synaptic dysfunction [28–31]. Since cerebral organoids harboring the E200K mutation do not develop spontaneous disease [28], this offers an opportunity to examine what events can initiate misfolding. Herein, we hypothesized that cellular stress may trigger PrP misfolding in organoids with E200K PrP. To test this, we used iPSCs generated from a donor with no known disease and an asymptomatic carrier of the E200K mutation. Both iPSC lines underwent CRISPR-Cas9 cloning to generate the three possible genotypes at codon 200 (E/E, E/K, K/K), for a total of six cell lines, which were used to generate cerebral organoids. Organoids were treated short and longer-term with 4-Hydroxynonenal (HNE), methylglyoxal (MGx) and HSV1 infection and monitored for misfolded PrP. Our data indicate that these stressors are insufficient to trigger PrPSc-like misfolding in *PRNP* E200K organoids.

## Materials and methods

### Ethics

The human cells used in this study were obtained from a commercial source (Applied Stem Cell) or from skin punch biopsy of an asymptomatic donor carrying a single (heterozygous) glutamic acid to lysine prion protein gene mutation at codon 200 (methionine homozygous at codon 129). These cells and procedures have been described previously [28,29]. Briefly, cells were collected at Case Western Reserve University following the Institutional Review Board (IRB) protocol IRB No. STUDY20181189 approved and monitored by University Hospitals Cleveland Medical Center and Case Western Reserve University School of Medicine. The informed consent form was signed and obtained from each donor. The skin punch biopsy samples were de-identified before being provided to the researchers at the NIH. Thus, the NIH Office of Human Subjects Research Protections (OHSRP) has determined these samples to be exempt from IRB review.

### Human iPSCs and cerebral organoids

#### iPSCs

Two sets of human induced pluripotent stem cell (iPSCs) lines were used to generate COs, one derived from a healthy donor and one derived from an asymptomatic donor carrying the E200K mutation. Both sets of cells were homozygous for methionine at codon 129. Our healthy, no-known disease line, ASE-9209, was purchased from Applied Stem Cell. Collection of cells from the E200K carrier donor and their reprogramming was described previously [28]. ACS-1023 (ATCC) and RAH019A iPSCs (described in [29]) were used to generate organoids for trial infections. Human iPSC lines were cultured on low growth factor Matrigel (Roche) in mTeSR1 medium (Stem Cell Technologies) with 5% CO2 in a humidified incubator. Media was changed every two days and colonies were passaged at approximately 80% confluence using ReLeSR (Stemcell Technologies) reagent as described in the manufacturer’s instructions.

#### CRISPR

CRISPR-Cas9 cloning was carried out by Applied Stem Cell using the following guide RNA (gRNA); PRNP-g2 GCGCTCCATCATCTTAACGTCGG. gRNAs and Cas9 were transfected into the iPSCs and positive cells selected by 48 hr incubation with puromycin. After selection a small population of cells underwent genotype analysis and single cell cloning by dilution to one cell per 200 μl. Cells were allowed to grow for 4 to 6 weeks before further genotype analysis. Two clones of each genotype were finally confirmed by NGS sequencing. The cloning strategy followed by Applied Stem Cell and the quality control results can be found in the supplementary materials of Wood & Foliaki et al. [32].

#### Cerebral organoids

COs were generated using the Cerebral Organoid Differentiation kit (Stemcell Technologies), which follows the protocol described by Lancaster and Knoblich [33]. Organoids were routinely cultured in complete maintenance media (1× glutamax, 1× penicillin/streptomycin solution, 0.5× non-essential amino acids, 0.5% [v/v] N2, 1% (v/v) B12 with retinoic acid, 1 μL/4 mL insulin, and 1 μL/286 mL 2-Merceptoethanol in 1:1 Neurobasal:DME-F12 medium), under standard incubator conditions (5% CO2, 37°C, humidified), on an orbital shaker at 85 rpm using vented conical flasks (Corning), as described in more detail in [30]. COs were used for experiments at ~4 months of age. Trial infections were done as described previously [30,34].

### Toxic compounds

A starting concentration curve of HNE and MGx was prepared to determine the optimal non-toxic dose (S1 Fig). PrestoBlue metabolic assays were done on COs prior to treatment with a range of concentrations of each compound, and then one week after treatment (one treatment on day zero, media changed on days three and five). Experimental concentrations were the highest concentration of each compound that did not cause significant changes in metabolism over the course of one week (HNE– 80 μM, Methylglyoxal– 5 mM). COs were maintained in maturation media. Media was changed twice weekly, and compounds added every other feeding.

### Viruses/Infections

#### Virus

Stocks of KOS-GFP and McKrae-GFP HSV Type 1 were produced and provided by William Halford from Southern Illinois University and are described in [35]. Wild-type viral strains were propagated in Vero cells cultured in complete DMEM (Dulbecco’s modified Eagle medium (DMEM) supplemented with 0.15% HCO_3_^−^, 10% fetal bovine serum, 100 U/ml penicillin G, and 100 mg/ml streptomycin). Viral titers were calculated and then virus stored at -80°C until use. Mock stocks consist of uninfected Vero cell lysate.

#### Cell counting

Cell counting was performed using the ‘Cell Count and Viability Assay’ as per instructions with the Muse cell counter (Luminex). In short, three random COs from each line were taken and dissociated gently using Accutase (Thermo Scientific). Lysate was diluted in ‘Muse Count and Viability Kit’ reagent (Luminex) and placed in the Muse for analysis. We averaged the total cell number across the three organoids to determine approximate number of cells per organoid.

#### MOI

We divided the average number of cells per organoid by stock concentrations of virus to determine the amount of virus solution to be added to each organoid for a multiplicity of infection (MOI) equal to 1. We then diluted by factors of 10 to create a range of MOIs to infect COs with. Equivalent amounts of Vero cell lysates were added to respective mock infections. To confirm viral infection, we monitored COs for GFP expression using the EVOS-FL-auto light microscope (Invitrogen).

#### Antivirals

COs were pretreated with 30 μM (*E*)-5-(2-bromovinyl)-2′-deoxyuridine (Brivudine) (Sigma-Aldrich) and 125 IU/ml Human Recombinant Interferon-α A2 (IFN- α) (StemCell Technologies) 24hr before infection to induce viral latency, and treatment was continued for 2 weeks to preserve viral latency [36]. Sporadic re-activation (GFP production) was monitored using the EVOS. The virus could be reactivated by heating the CO at 45°C for ten minutes, emulating a fever [37]. Brivudine was reconstituted in methanol and IFN-α was reconstituted in sterile water. Both were added to maintenance media on the day of use and stored long term in -80°C and short term in -20°C.

### Immunofluorescence

COs were fixed in 10% (w/v) neutral buffered formalin (NBF) for at least 2 days, shaking at room temperature (RT). They were washed in 1× PBS and then allowed to equilibrate in 20% and 30% (w/v) sucrose solutions, shaking at RT. COs were then coated with a blue dye for visualizing tissue, embedded in OCT (Ted Pella, Inc.) and stored at -20°C until use. COs were sectioned into 10 micron slices using a cryostat (Leica), mounted on superfrost slides (Thermo Scientific) and stored in 4°C until staining. After removing OCT, tissue was rehydrated and washed with 1× PBS. Tissue was incubated for 1 hour at RT in blocking solution (5% (w/v) BSA, 0.3 M glycine, and 0.1% Triton X-100 in 1× PBS) to prevent unspecific binding and permeabilize cells, then rinsed in 1× PBS, incubated for 5 min with 1:1000 Dapi (Invitrogen), and rinsed again in 1× PBS. Slides were treated with Prolong gold (Invitrogen), mounted with a coverslip and allowed to cure overnight at RT. Images were collected using the EVOS. GFP produced by the virus was preserved throughout the staining process.

### PrestoBlue

Cell metabolism was measured using PrestoBlue Cell Viability Reagent (Thermo Scientific) according to the manufacturer’s instructions. In short, reagent was diluted 1 in 10 in warm organoid media. Organoids were cultured in 24 well plates, with one organoid per well. Existing media was removed and replaced with 500 μls per well of made-up PrestoBlue media. The plates were returned to the shaking incubator for 30 minutes. The metabolized PrestoBlue media was then removed and placed into 3 replicate wells at 150 μL aliquots for analysis. Fluorescence was measured using a ClarioStar plate reader (BMG) at 560 nm excitation and 590 nm emission.

### PK digests, detergent insolubility and western blotting

COs were homogenized to 10% (w/v) in RIPA Lysis and Extraction buffer (25 mM Tris HCl pH 7.6, 150 mM NaCl, 1% NP-40, 1% sodium deoxycholate, 0.1% SDS: Thermo Scientific) or phosphate buffered saline (PBS; 10 mM phosphate, 150 mM sodium chloride, pH 7.4) by motorized pestle, cleared with a 2 minute 2000× centrifugation, and stored at -80°C until use. For protease digest, CO homogenates were thawed and 13.5 μL of each sample were added to a tube with 1.5 μL of 50 μg/ml Proteinase K (PK) in 10% Sarkosyl for a final concentration of 5 μg/ml PK and 1% Sarkosyl. Solutions were incubated for 1 hour at 37°C with 400 rpm shaking. Five μL of 4× Bolt LDS sample buffer (Invitrogen) containing 6% (v/v) β-mercaptoethanol was added to each sample and samples were boiled for 5 min. For total PrP controls, 5 μL of 10% brain homogenate was added to 10 μL of PBS and 5 μL of Bolt LDS sample buffer and boiled for 5 minutes. The detergent insolubility protocol has been described previously [28]. Briefly, lysates were treated with 300 μL of 10% (w/v) sarkosyl for 1 h at RT with mixing at 1400 rpm, then diluted into 2680 μL of H-Buffer (10 mM Tris–HCl, 1 mM EGTA, 0.8 M NaCl and 10% sucrose at pH 7.4), and centrifuged at 100,000× g for 1 h at 4°C. The pellets were resuspended in 1× sample buffer for Western blot analysis. The proteins in the supernatants were precipitated by a 5 fold excess of ice cold methanol with overnight incubation at -20˚C before centrifuging at 20,000×g for 1 h and the pellet resuspended in 1× sample buffer for Western blot analysis. Samples were boiled for 5 minutes. All samples were centrifuged briefly before loading into a gel to remove condensation. Twenty μL of each sample were loaded into wells of Bolt 4–12% Bis-Tris gels (Invitrogen) and electrophoresis carried out at 200V for approximately 20 minutes. Gels were transferred to PVDF membranes using the iBlot 2 transfer system (Invitrogen). Membranes were blocked in 5% milk in tris-buffered saline (20mM [2.42g/L] tris base and 0.15M [8.77g/L] sodium chloride, pH 7.5) with 0.05% Tween (TBST) for 30 minutes on a rotating shaker. Primary antibody 3F4 (Millipore) to detect PrP was added at a 1:10,000 dilution in 5% blocking solution at 4°C overnight. Secondary antibody (Abcam) was added at 1:5,000 in blocking solution for 2 hours room temperature. ECL Select (Amersham) was used to visualize protein bands with images collected using the iBright imaging system (Invitrogen).

### RT-QuIC

Real-time quaking-induced conversion (RT-QuIC) assays to measure prion seeding activity were performed as previously reported [28,34]. In brief, COs were homogenized to 10% (w/v) in PBS by motorized pestle, cleared with a 2-minute 2000×g centrifugation, and stored at -80°C until use. After thawing, samples were serially diluted by 10 folds in 0.1% sodium dodecyl sulfate (SDS)/PBS/N2. Each sample was tested in quadruplicate wells of a 384 well plate (Nunc). One μL of CO dilutions was added to each well along with 49 μL of reaction mix (final concentrations of: 10 mM phosphate buffer [pH 7.4], 300 mM NaCl, 0.1 mg/mL hamster recombinant PrP 90–231, 10 μM thioflavin T (ThT), 1 mM ethylenediaminetetraacetic acid tetrasodium salt and 0.002% (w/v) SDS. Plates were sealed and incubated in a FLUOstar Omega plate reader (BMG) at 50°C for 50 hours with cycles of 60 s of shaking (700 rpm, double orbital) and 60 s of rest. ThT fluorescence was measured every 45 minutes (excitation, 450 ± 10 nm; emission, 480 ± 10 nm [bottom read]). Wells were considered positive for seeding activity when fluorescence exceeded a threshold of 10% of the maximum value from any individual reaction wells on each plate within the 50h time cutoff. A sample was considered positive if greater than 25% of the reaction wells showed positive seeding activity. Data was plotted using GraphPad Prism 9.

### Image quantification and statistical analyses

Image files saved using the EVOS imaging system were analysed using Fiji Image J 1.52n software. For each image, a region of interest was drawn around each organoid, with a second background region selected away from the organoid. The mean intensity of the background region was subtracted from that of the organoid region of interest. Plots were generated and statistical analyses performed using Graphpad Prism 8.2.0. The statistical test used is indicated in the figure legend.

## Results

**Fig 1:**
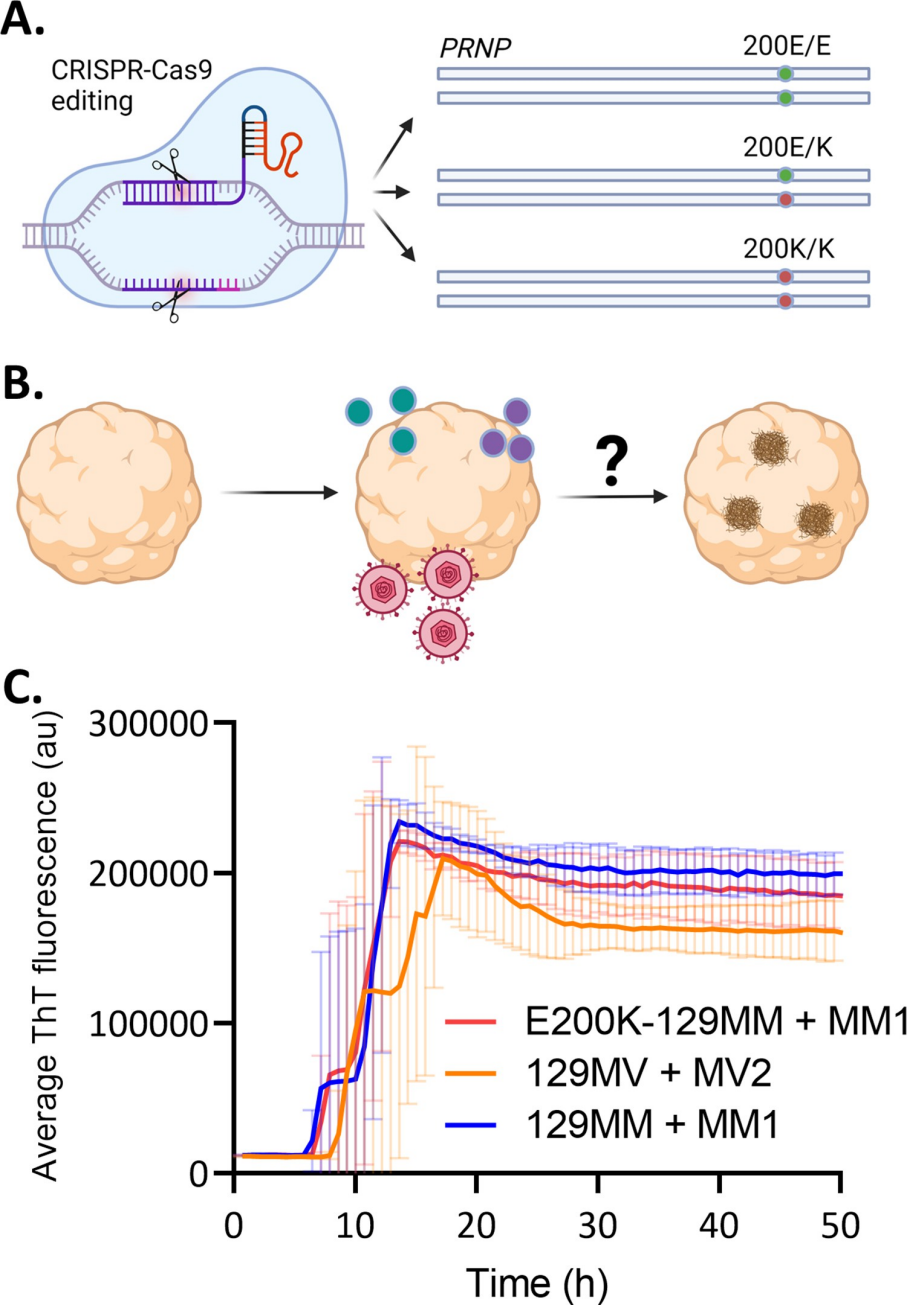
Experimental plan. **A.** Schematic showing the CRISPR-Cas9 engineering of all three genotypes at codon 200 (E/E–isotype control, E/K heterozygous mutation, K/K homozygous mutation). **B.** Diagram of the experimental plan to grow the organoids until they contain mostly mature cells (around 4 months old) and add to their media HNE or MGx (green/purple dots) or HSV1 (pink virus) and monitor for the emergence of misfolded species (brown deposits). Created with Biorender. **C.** Representative RT-QuIC fluorescence traces (showing the mean and standard deviation of 4 replicate reactions per test condition) from sCJD MM1-infected E200K-129MM organoids as compared with MM1 sCJD-infected 129MM and MV2 sCJD-infected 129MV control organoids at >150 dpi.

COs from donors carrying the PRNP E200K mutation that causes genetic CJD do not show production of spontaneous prions [28]. This provides an opportunity for investigating what stressors might trigger prion misfolding and propagation. We hypothesized that cellular insults including those associated with oxidative stress and viral infection might be sufficient to trigger misfolding. To test this, we used CRISPR-Cas9 engineering to make isogenically matched *PRNP* 200E/E, 200E/K and 200K/K induced pluripotent stem cells (iPSCs; Table 1, Fig 1A) from both an E200K carrier donor and a no-known disease (NKD) control donor. The isogenically matched cells allow us to assess any dose contribution by the K allele and provide the most appropriate controls (200E/E in the same genetic background as 200E/K or 200K/K). Organoids were differentiated from each of these iPSC lines and cultured until they were mostly populated with maturing neurons, astrocytes and oligodendrocytes (~4 months old) then exposed to the cellular insults. After this they were monitored over short and longer periods of time to look for the emergence of misfolded species (a simplified experimental plan is shown in Fig 1B). Prior to beginning the treatments, we confirmed that our E200K donor organoids were capable of propagating prions by infecting them with brain homogenate from a person who died of MM1 sCJD. Example Real-Time Quaking Induced Conversion (RT-QuIC) traces are shown in Fig 1C compared with organoids from two unrelated iPSC lines that also propagate prions from human brain homogenate (RT-QuIC positive MM1 E200K PrP is not detectable following PK digest but demonstrates increased insolubility; S1 Fig). This shows that, while the E200K organoids do not spontaneously produce prion propagation, they can propagate prions from an initiating event, which can be detected by RT-QuIC.

**Table 1:** iPSC CRISPR-Cas9 engineered lines.

Donor line			CRISPR-Cas9 engineered *PRNP* 200 genotypes	
Name	*PRNP* 129 genotype	*PRNP* 200 genotype		
E200K donor	M/M	E/K	E/E	K/K
No Known Disease (NKD) donor	M/M	E/E	E/K	K/K

### HNE and MGx cellular stressors do not induce E200K PrP misfolding

**Fig 2:**
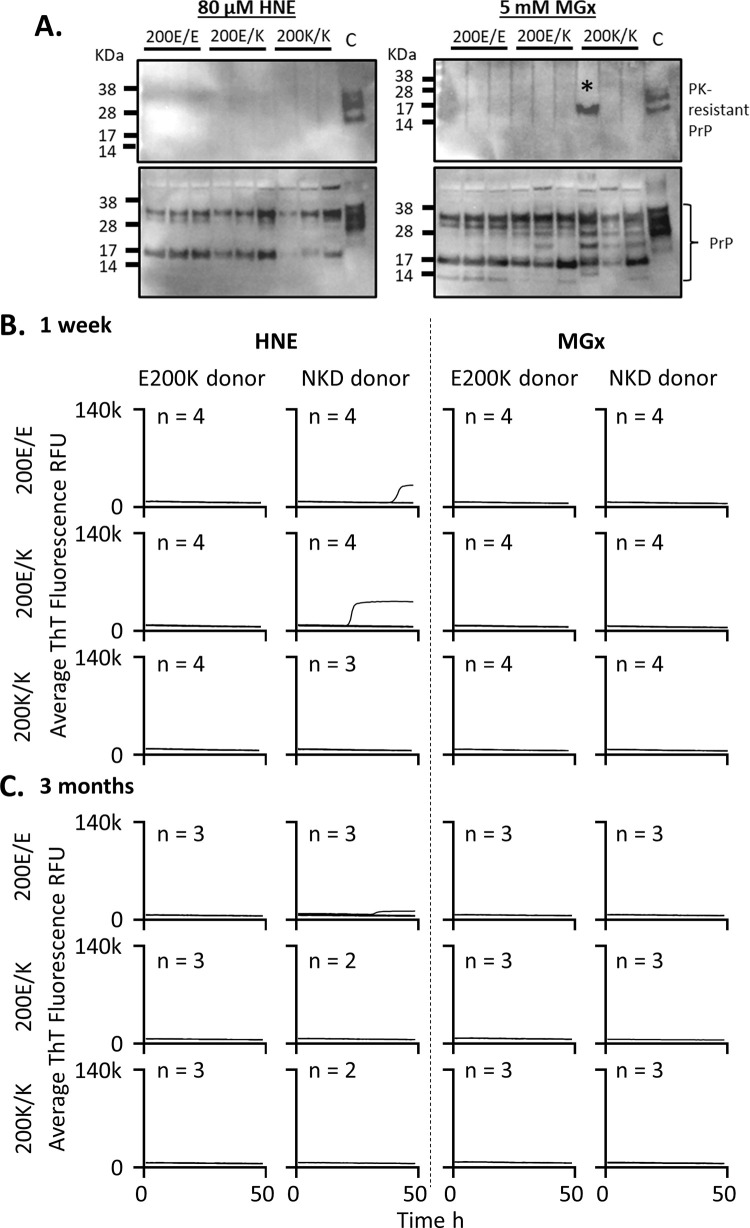
HNE and MGx oxidative insults do not cause PrP misfolding or accumulation of seeding activity after short- and longer-term exposure. **A.** Western blots of the E200K donor lines one week following exposure to HNE and MGx (3F4 primary antibody). Shown are protease (PK) digested PrP as compared with sCJD PK digested brain tissue (upper) and undigested PrP compared with undigested sCJD brain homogenate (lower) from the E200K donor E/E, E/K and K/K genotype organoids. Numbers above lanes indicate individual organoids, *denotes band likely to be due to incomplete digestion. **B & C**. RT-QuIC assays for all genotypes and both treatments at 1 week (B) of treatment and after (C) 3 months of weekly treatments. Traces shown are averages of 4 replicate reactions per ‘n’ individual organoids, with the ‘n’ indicated on each graph. NKD = no-known disease donor. None of the samples exceeded the 25% positive well threshold to be considered positive. Control reactions are shown in S4 Fig.

As cells age or suffer stress insults, toxic byproducts of metabolism are formed. We first tested whether two such metabolites 4-hydroxy-2-nonenal (HNE), a product of lipid peroxidation, and methylglyoxyal (MGx), a toxic by-product of glycolysis, would cause E200K PrP misfolding. Cells were treated once weekly with concentrations of the insults that were below the toxicity threshold (S2 Fig). At 1 week post initial insult and 3 months of weekly insults, organoids were harvested and analyzed for the presence of misfolded PrP. To determine if PrP misfolding was occurring two assays were used; western blotting after protease digest and RT-QuIC, which detects prion seeding activity that is indicative of misfolded prions. For protease digestion before western blotting, a low quantity of proteinase K (PK: 5 μg/ml) was used to detect small levels of resistance that might occur with small or early structural changes. After one week of treatment, detection of protease resistant PrP was present only in one lane of the E200K donor organoids engineered to be homozygous for the K/K mutation that were treated with MGx (Fig 2A, *indicates band). A noticeable change was observed in the banding pattern of the undigested PrP, indicative of increased cleavage or fragmentation of the protein (Fig 2A; see S3 Fig for examples of the unstressed organoid PrP banding pattern). No seeding activity as detected by RT-QuIC was present at one week following treatment (Fig 2B). Likewise, after 3 months of weekly treatments, no seeding activity had emerged (Fig 2C). This indicates these insults were ineffective in initiating prion misfolding and seeding that produces on-going misfolded forms.

### Acute HSV1 infection of E200K cerebral organoids does not initiate PrP misfolding

**Fig 3:**
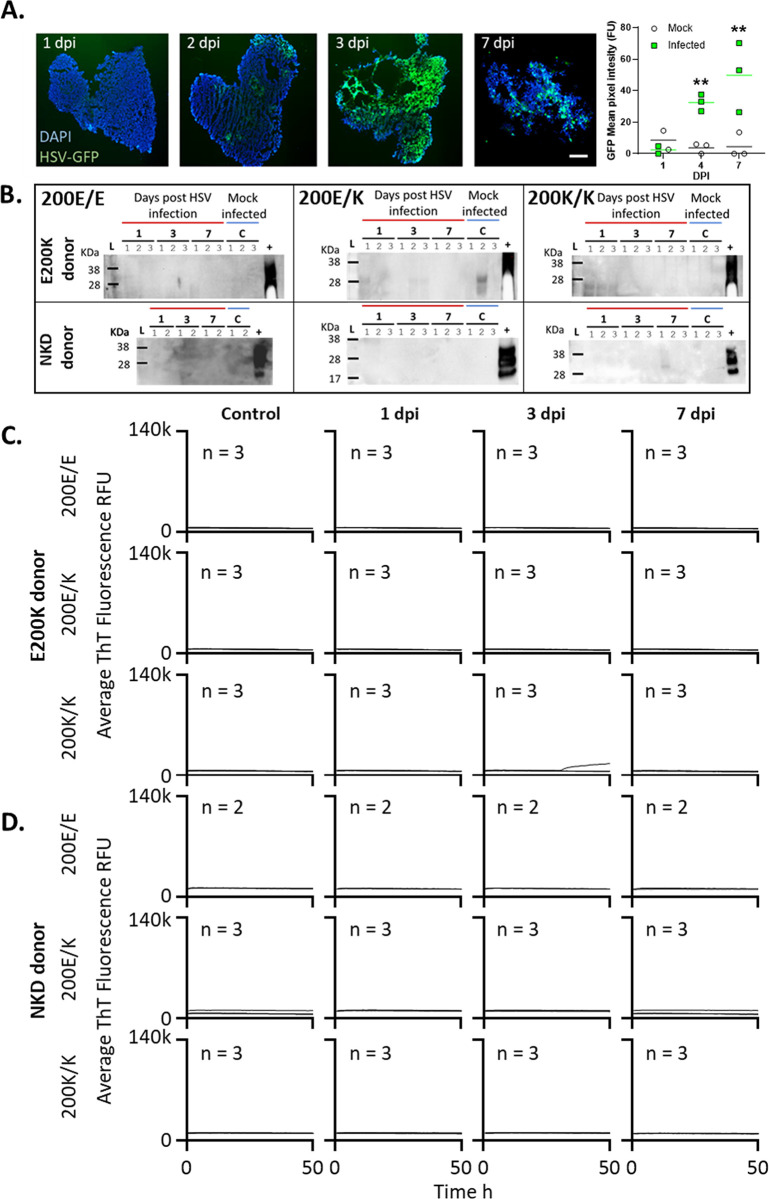
Acute HSV1 infection does not cause production of misfolded PrP or prion seeding activity accumulation. Organoids were infected with a 0.00001 MOI of McKrae HSV1. **A.** Emergence of HSV1-GFP over 1–7 dpi demonstrating active viral replication and changing organoid morphology by 7 dpi. Scale bar = 500 μm. Quantification of GFP fluorescence in organoids over time indicating production of virus is shown right. Individual points show single organoids (n = 3) and horizontal lines show the average intensity. **p<0.01 from the background fluorescence of mock-infected organoids determined by two-way ANOVA with Tukey’s secondary testing. **B.** Protease-resistant PrP at 7 dpi compared with sCJD positive control brain homogenate (+). Numbers above lanes indicate individual organoids. **C & D.** RT-QuIC seeding activity assays of the E200K donor organoids (C) and no-known disease (NKD) donor organoids (D) from 1–7 dpi compared with uninfected control organoids. Control organoids were assayed at 7 days post ‘mock’ infection with Vero cell lysates. Traces shown are averages of 4 replicate reactions per ‘n’ individual organoids, with the ‘n’ indicated on each graph. None of the samples exceeded the 25% positive well threshold to be considered positive.

As the oxidative stress insult alone did not result in PrP misfolding, we hypothesized that a more complex series of cellular insults would be needed to destabilize E200K PrP. A more substantial insult is provided by neurotropic viral infection such as HSV1, which has been demonstrated to infect human COs [36,38,39]. Therefore, we selected this virus to investigate whether it could cause protein misfolding of E200K PrP. Initially, we infected organoids with two strains of HSV1 to look for protein misfolding in response to the actively proliferating virus. Both strains of HSV1 were tagged with GFP allowing us to monitor the presence of actively replicating virus by fluorescence imaging (Fig 3A & 3B). Of the two strains and six multiplicities of infection (MOI) tested, only organoids infected with the McKrae strain at the 0.00001 MOI showed viability at one week by Prestoblue assay of their metabolism (S5 Fig). This was the concentration chosen to assess the changes in PrP, however, even at this concentration, organoids looked morphologically stressed with less defined edges and greater cell debris observed after 1 week (Fig 3A) and all infected organoids were dead by three weeks (S5 Fig). Organoids were harvested at 0, 1, 3 and 7 dpi to look for protease resistant PrP or changes in protein seeding activity. None of the genotypes from either the E200K donor or the NKD donor organoids showed any evidence of protease resistant PrP (Fig 3C) apart from a couple of faint bands, which likely represented incomplete digest at the low levels of PK enzyme (as the strongest of these was observed in the E200K E/K organoid control condition). In contrast with the HNE and MGx treatments no changes were seeing in PrP banding profile of undigested PrP (S3 Fig). RT-QuIC seeding activity was also absent (Fig 3D & 3E). Thus, 7 days of acute HSV1 infection was insufficient to cause any E200K PrP misfolding.

### Latent HSV1 infection does not result in PrP misfolding in E200K cerebral organoids

**Fig 4:**
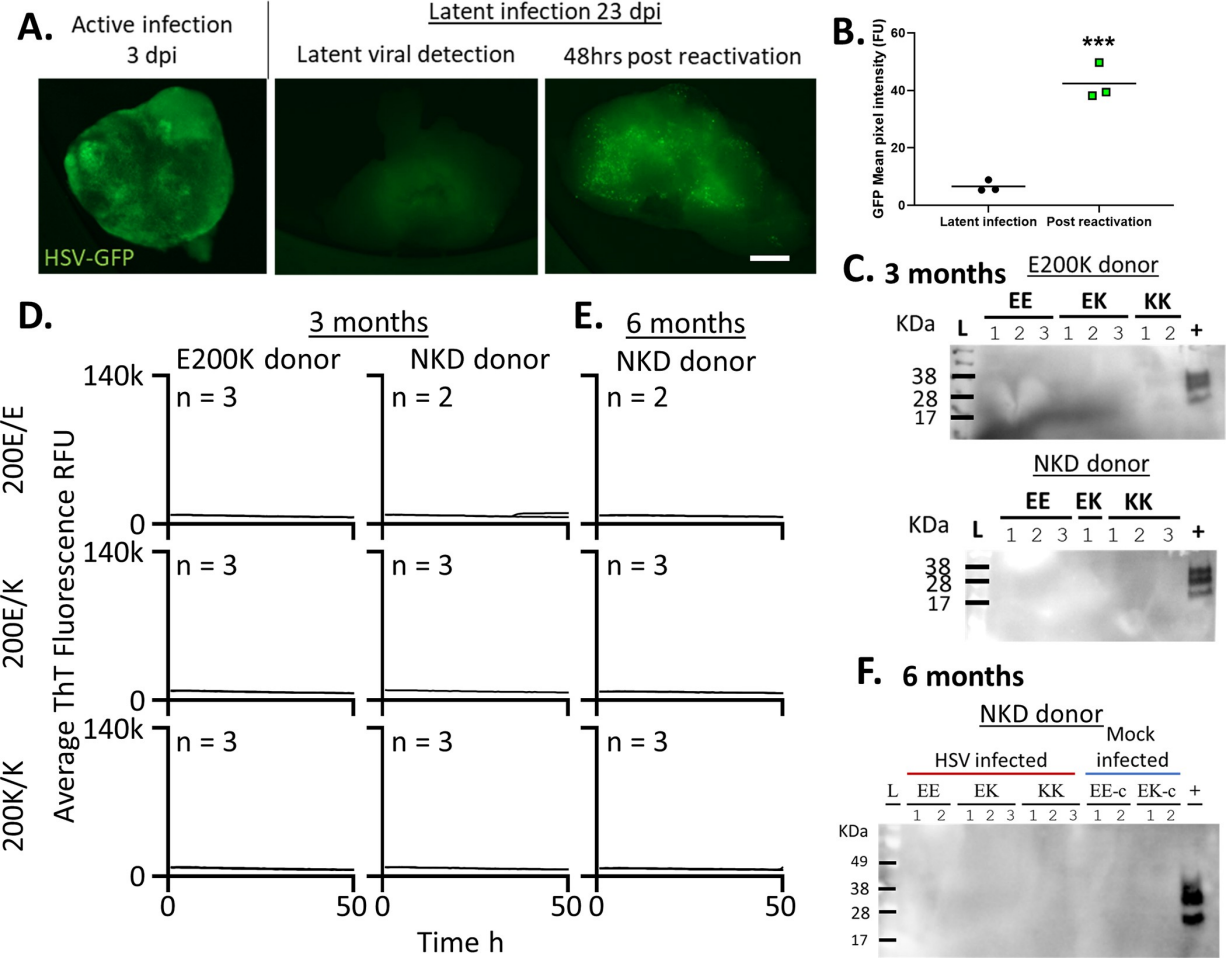
Latent HSV1 infection does not result in emergence of PrP misfolding in the E200K PrP organoids. Organoids were infected with a 0.00001 MOI of McKrae HSV1 while treated with IFNα and brivudine from 24 hr before infection to 14 dpi. **A.** Example live organoid images of active infection at 3 dpi and latent infection at 23 dpi with or without reactivation by heat shock. Scale bar = 500 μm. **B.** Quantification of GFP signal following reactivation of HSV1 replication. Individual points show single organoids (n = 3) and horizontal lines show the average intensity. ***p<0.001, Student’s t-test. **C.** Protease resistant PrP detection in organoids at three months of latent infection compared with sCJD positive control brain homogenate. **D.** RT-QuIC seeding activity assays at 3 months post infection and **E.** at 6 months post-infection for the NKD donor. Traces shown are averages of 4 replicate reactions per ‘n’ individual organoids, with the ‘n’ indicated on each graph. None of the samples exceeded the 25% positive well threshold to be considered positive. **F.** Protease resistant PrP at 6 months of latent infection for the NKD donor compared with positive control sCJD homogenate (+). Numbers above lanes indicates individual organoids. Control organoids (age-matched, treated with compounds and ‘mock’ Vero cell lysates) can be found in S7 Fig.

As the viability of the HSV1 infected organoids declined rapidly we could not monitor the effect of long-term active HSV1 infection on prion misfolding. To examine organoids infected over a longer period of time we used a latent infection system. While we trialed a number of anti-viral strategies for inducing latent infection (S6 Fig), the system developed by D’Aiuto et al. [36] appeared to produce consistent latent infection that did not spontaneously reactivate but could be readily reactivated by heat shock treatment of the organoids (Fig 4A & 4B). Organoids were treated for 24 hours with IFNα and Brivudine then infected with HSV1, and antiviral treatments were continued for a further 2 weeks after which the organoids were maintained in standard media. Organoids remained viable (and of healthy appearance) throughout the entire infection period. By three months of infection no protease resistant PrP (Fig 4C) and no changes in seeding activity (Fig 4D) were observed in either organoid set. We also followed the NKD donor lines out to six months post infection and did not observe any further changes (Fig 4E & 4F). Altogether, we conclude these insults were not sufficient to induce destabilizing changes in E200K PrP in human cerebral organoids.

## Discussion

In this study, we sought to use the lack of abnormal PrP in cerebral organoids with the CJD-associated *PRNP* E200K mutation to ascertain whether certain cellular insults could trigger E200K PrP misfolding. For this, we treated E200K organoids (and their respective controls) with 4-Hydroxy-2-nonenal or Methylglyoxal, two byproducts of cellular stress, or infected them with neurotropic HSV1. Our data show that none of these insults was sufficient to induce changes in E200K PrP that might be associated with protein misfolding in human cerebral organoids. While we cannot conclude that these insults do not trigger PrP misfolding and disease onset in human adults, our data leads us to believe that other factors must come into play. We did however confirm our previous results that found no protease-resistant or seeding-active PrP formed spontaneously in the E200K organoids [28] and showed this is true even when both 200 alleles are lysine.

The only protease-resistant band observed in this series of experiments was in the 200K/K donor cells treated with MGx at 1 week of treatment. This had an unusual appearance, with a single band rather than three bands, and was detected at low PK levels (for comparison 100 μg/ml solutions, 20× more PK than used herein, are used to digest human brain for molecular subtyping [40]). We cannot preclude the possibility that this band represents a structural modification that is an intermediary species occurring prior to more substantial misfolding that results in on-going propagation. However, given the lack of seeding activity at this time and after 3 months incubation, it is most likely that this band represents an incomplete digest. Of interest was the changed banding pattern of the undigested PrP in HNE and MGx as this indicated the treatments were sufficient to affect the organoids. Increased cleavage or fragmentation of PrP is associated with oxidative stress [41–43] and, therefore, this banding pattern indicated that the oxidative insults were having the expected direct effect on PrP itself. However, this was not relevant to its misfolding as neither short-term RT-QuIC seeding activity was positive nor an emergence of misfolded species over a longer term was detected.

Neurotropic viruses cause significant stress, damage, and death within human cerebral organoid cultures [36,38,39,44]. A recent study reported PrP misfolding in neuroblastoma cells after infection with influenza A virus [45]. The misfolded prions produced were infectious and transmissible to mice causing death around 177 dpi with deposition of PrP, although the disease was distinct from mouse scrapie strains. HSV1 is a common neurotropic virus. Symptoms of the initial infection are typically mild but, after acute infection, HSV travels retrograde up neural axons and establishes life-long latent infection in neurons [46]. Interestingly, intrathecally synthesized antibodies against HSV have been detected in cerebrospinal fluid from a small number of CJD patients [25] and active virus has been found in two CJD patients [26]. PrP^C^ expression has been linked with promoting HSV1 replication in mice [47,48]. Conversely, an anti-viral role of PrP^C^ was identified in astrocytes through positive regulation of autophagy [49]. Given the links between viral infections and PrP, we tested the potential of this virus to destabilize E200K PrP causing misfolding. As with the individual oxidative insults no effect of infection, acute or latent, was observed. However, it remains possible that multiple reactivation events could be necessary to put the brain under enough pressure to trigger conversion, but we did not investigate such a possibility herein. In addition, cerebral organoids do not contain all brain cell types—they are lacking microglia and endothelial cells, which may be critical in the initial prion misfolding events. While HSV1 may not cause the initial misfolding of PrP, it is still possible that it or other neurotropic viruses might influence the onset, spread and pathogenesis of prion diseases [50]. Indeed, a recent case study found accelerated prion disease in a patient infected with Sars-Cov-2 [51]. Overall, HSV1 infection in this human brain model is not an event that causes E200K PrP misfolding.

## Conclusion

Herein we have shown that oxidative stress insults and HSV1 infection, acute or latent, do not cause early destabilization and misfolding of E200K PrP or emergence of a misfolded species over a longer time in culture. We cannot conclude that these factors are not involved in the events that lead to the propagation of prions within the human brain, but they are insufficient to cause such changes to occur in human cerebral organoids with E200K PrP.

## Supporting information

S1 Fig Western blotting showing accumulation of insoluble PrP in the MM1 infected E200K organoids. Control and E200K organoids were infected with MM1 sCJD brain homogenate and collected at 100 and >150dpi respectively. In similarity with our previous results in control organoids infected with MV1, the MM1 infected control and E200K organoids did not demonstrate protease resistance [30] but detergent insoluble species were present. Insoluble PrP is not found in organoids in the absence of RT-QuIC positivity and was previously shown not to occur spontaneously in aged uninfected E200K organoids [28].
(PDF)

S2 Fig HNE & MGx toxicity curves. Titrations of HNE and MGx were done on organoid cultures and the highest concentration that did not produce a decrease in cellular metabolism (Prestoblue assay) indicative of reduced viability was used for experiments. Dots show individual organoids with the mean indicated as a bar. One-way ANOVA with Tukey secondary testing was used to identify significant reductions from control organoids. ***p<0.001, **p<0.01.
(PDF)

S3 Fig Western blot analysis of HSV infected and control organoids for undigested PrP. Western blotting of the E200K donor isogenic organoids at 1, 3, and 7 days of acute infection with a 0.00001 MOI of McKrae HSV1 and control organoids. No change in PrP banding patten is seen from controls. PrP reduction at 3 & 7 dpi is likely a result of viral toxicity. C = brain homogenate control, L = molecular weight ladder.
(PDF)

S4 Fig HNE & MGx RT-QuIC control data. Untreated control organoids were collected at the same timepoints as the test organoids for RT-QuIC analysis, and sCJD infected organoids were included as a positive control. **A.** Examples of sCJD (positive) and normal brain homogenate (NBH; negative) infected organoid control reactions. **B & C.** Control isogenically matched organoids collected at (B) one week and (C) 3 months of HNE and MGx treatment. Traces shown are averages of 4 replicate reactions per ‘n’ individual organoids, with the ‘n’ indicated on each graph.
(PDF)

S5 Fig HSV toxicity assessment by Prestoblue metabolism. Organoid viability when infected with a MOI 10-fold serial dilution of Kos and McKrae HSV1 strains 7 days post infection (dpi) and 0.00001 MOI of McKrae at 20 dpi. Dotted lines indicate the day 0, 100% control viability for the organoids in each condition. One-way ANOVA with Tukey secondary testing was used to compared changes in the different MOIs from the mock controls at 7 dpi and one-sample student’s t-testing compared the change in viability of the 0.00001 McKrae HSV1 strain at 20 dpi from its day 0 100% control. ***p<0.001.
(PDF)

S6 Fig HSV latency treatment viability assays. Viability of organoids (Prestoblue metabolism assay) after infecting with 0.00001 MOI McKrae HSV1 for (**A**) 7 days and (**B**) three weeks with the indicated antiviral treatments either started 24 hrs before infection and continued for 15 days (to 14dpi) or started at 3 dpi and continued to 14 dpi. Dots show individual organoids and means are indicated by bars. One way ANOVA analysis with Tukey’s secondary testing, comparing viability of the treatments with the untreated infected control, showed no significant changes in viability at 7 days (A) and at 3 weeks (B) only the Brivudine and Acyclovir treatments co-administered with interferon-α 24 hrs prior to infection remained significantly viable.
(PDF)

S7 Fig Latent HSV infected RT-QuIC control data. Untreated control organoids were collected at the same timepoints as the test organoids for RT-QuIC analysis, and sCJD infected organoids were included as a positive control. **A.** Control isogenically matched organoids collected at 3 and 6 months of latent HSV1 infection. Traces shown are averages of 4 replicate reactions per ‘n’ individual organoids, with the ‘n’ indicated on each graph. **B.** Example sCJD (positive) and normal brain homogenate (NBH; negative) infected organoid control reactions.
(PDF)

S1 Raw images (PDF)
