# Supplementary figures and images for: Stress and viral insults do not trigger E200K PrP conversion in human cerebral organoids
Source: PLoS One. 2022 Oct 27;17(10):e0277051. doi: 10.1371/journal.pone.0277051 (PMC9612459; doi:10.1371/journal.pone.0277051)

**S1 Fig**

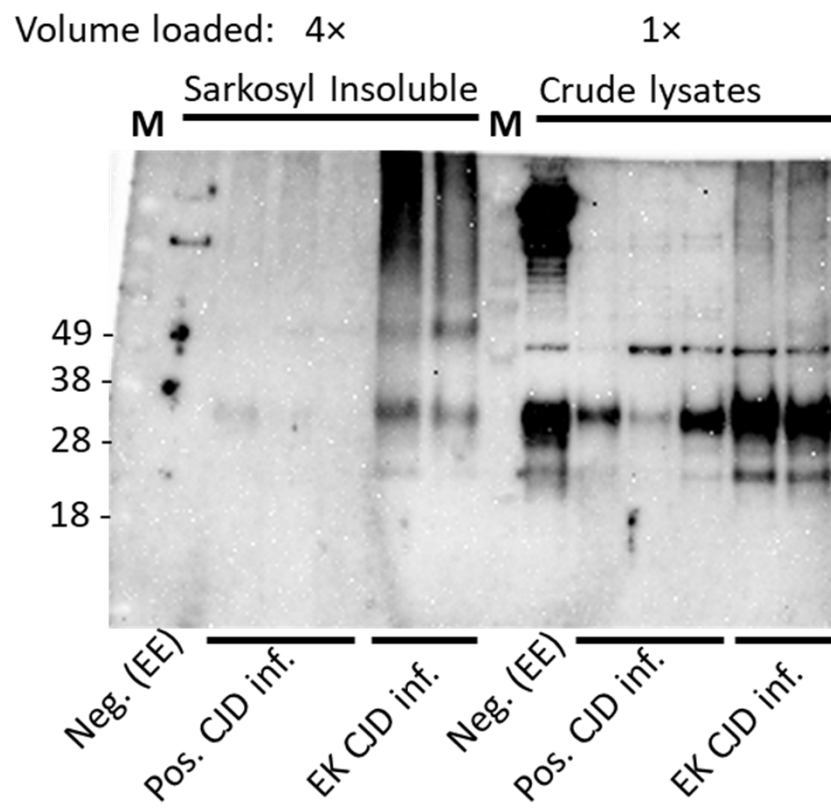

Supplement: S1 Fig — Control and E200K organoids were infected with MM1 sCJD brain homogenate and collected at 100 and >150dpi respectively. In similarity with our previous results in control organoids infected with MV1, the MM1 infected control and E200K organoids did not demonstrate protease resistance [30] but detergent insoluble species were present. Insoluble PrP is not found in organoids in the absence of RT-QuIC positivity and was previously shown not to occur spontaneously in aged uninfected E200K organoids [28]. (PDF) [file pone.0277051.s001.pdf]

S2 Fig

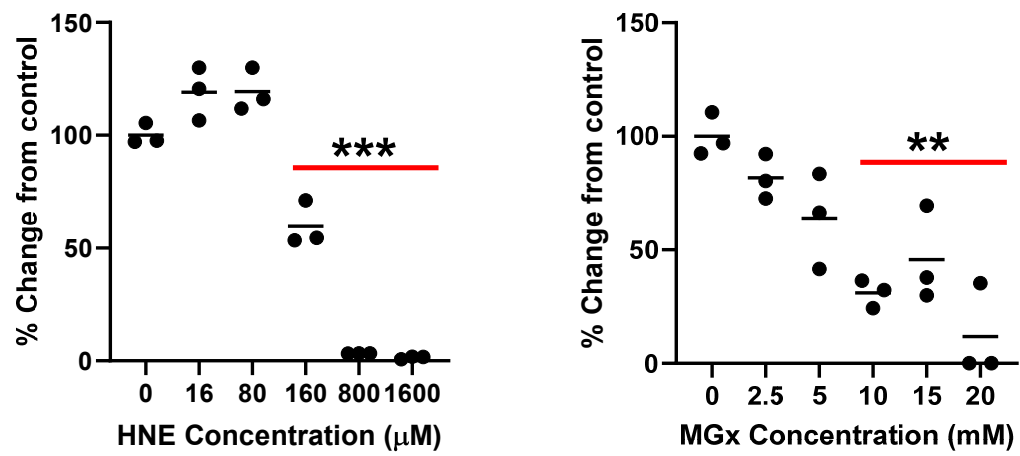

Supplement: S2 Fig — Titrations of HNE and MGx were done on organoid cultures and the highest concentration that did not produce a decrease in cellular metabolism (Prestoblue assay) indicative of reduced viability was used for experiments. Dots show individual organoids with the mean indicated as a bar. One-way ANOVA with Tukey secondary testing was used to identify significant reductions from control organoids. ***p<0.001, **p<0.01. (PDF) [file pone.0277051.s002.pdf]

S3 Fig

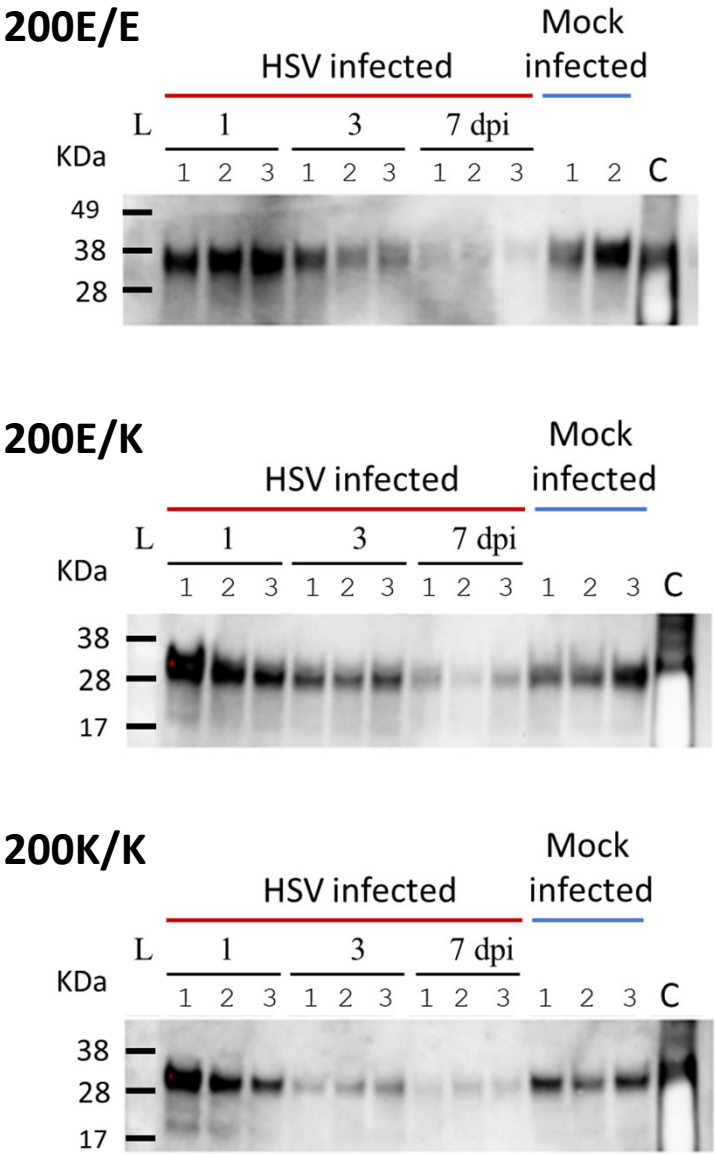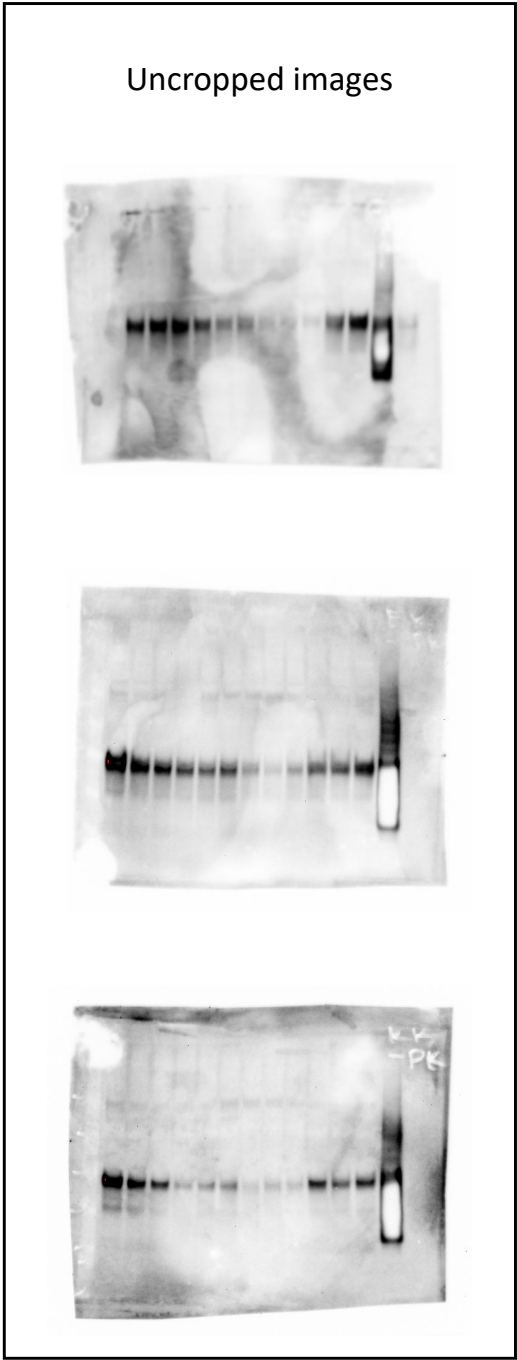

Supplement: S3 Fig — Western blotting of the E200K donor isogenic organoids at 1, 3, and 7 days of acute infection with a 0.00001 MOI of McKrae HSV1 and control organoids. No change in PrP banding patten is seen from controls. PrP reduction at 3 & 7 dpi is likely a result of viral toxicity. C = brain homogenate control, L = molecular weight ladder. (PDF) [file pone.0277051.s003.pdf]

S4 Fig

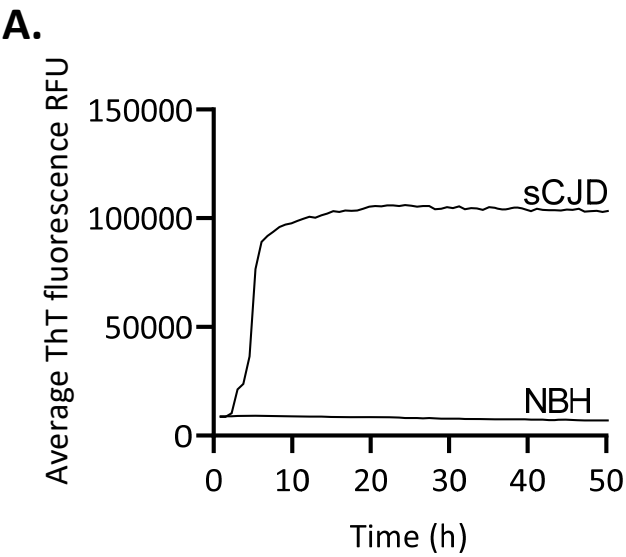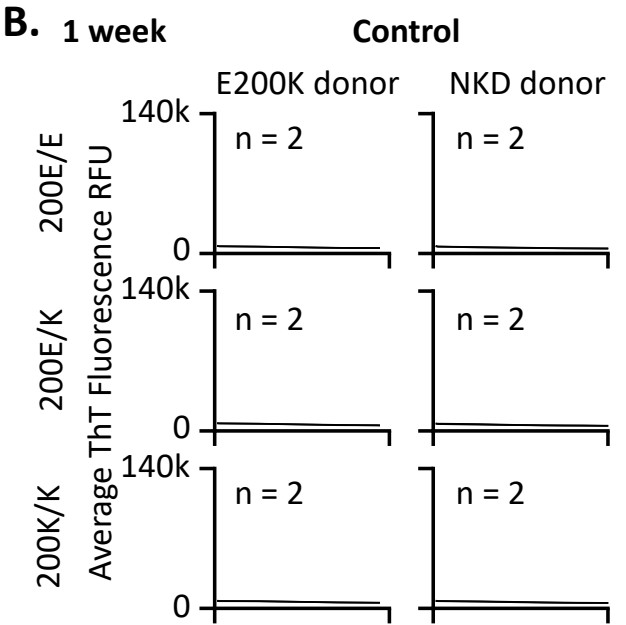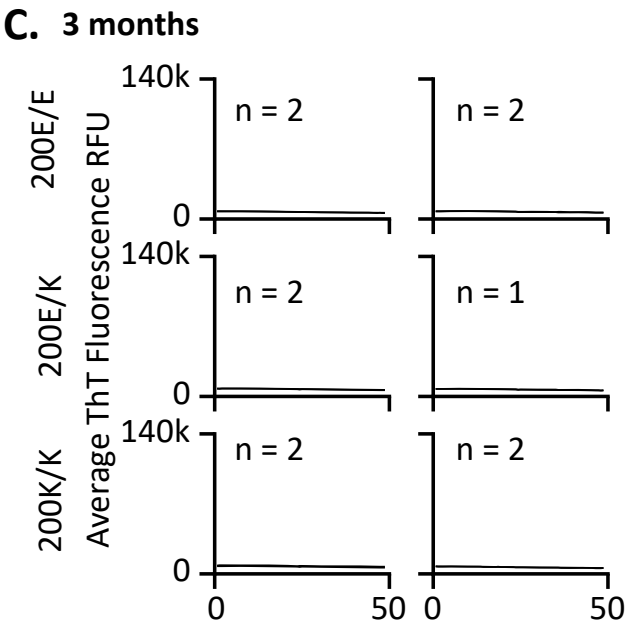

Supplement: S4 Fig — Untreated control organoids were collected at the same timepoints as the test organoids for RT-QuIC analysis, and sCJD infected organoids were included as a positive control. A. Examples of sCJD (positive) and normal brain homogenate (NBH; negative) infected organoid control reactions. B & C. Control isogenically matched organoids collected at (B) one week and (C) 3 months of HNE and MGx treatment. Traces shown are averages of 4 replicate reactions per ‘n’ individual organoids, with the ‘n’ indicated on each graph. (PDF) [file pone.0277051.s004.pdf]

S5 Fig

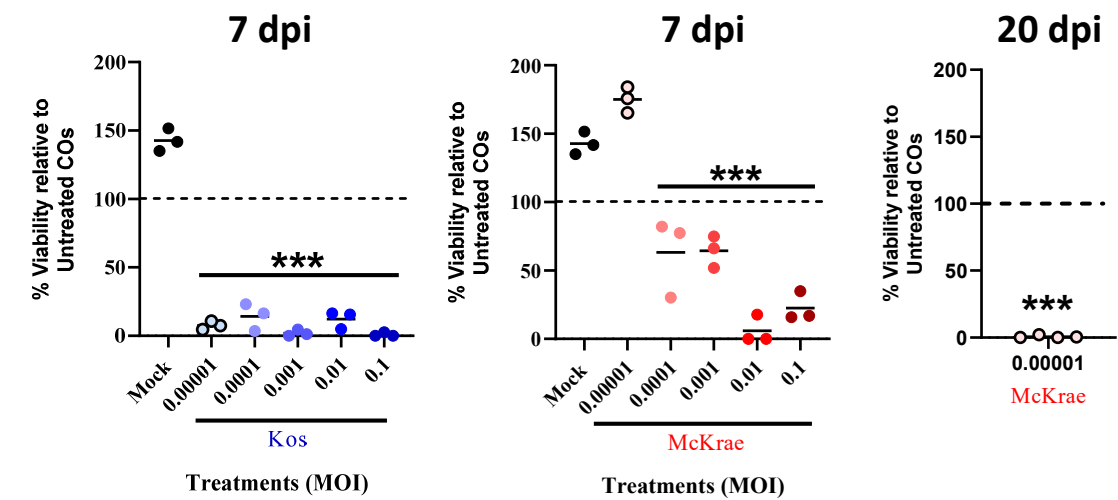

Supplement: S5 Fig — Organoid viability when infected with a MOI 10-fold serial dilution of Kos and McKrae HSV1 strains 7 days post infection (dpi) and 0.00001 MOI of McKrae at 20 dpi. Dotted lines indicate the day 0, 100% control viability for the organoids in each condition. One-way ANOVA with Tukey secondary testing was used to compared changes in the different MOIs from the mock controls at 7 dpi and one-sample student’s t-testing compared the change in viability of the 0.00001 McKrae HSV1 strain at 20 dpi from its day 0 100% control. ***p<0.001. (PDF) [file pone.0277051.s005.pdf]

S6 Fig

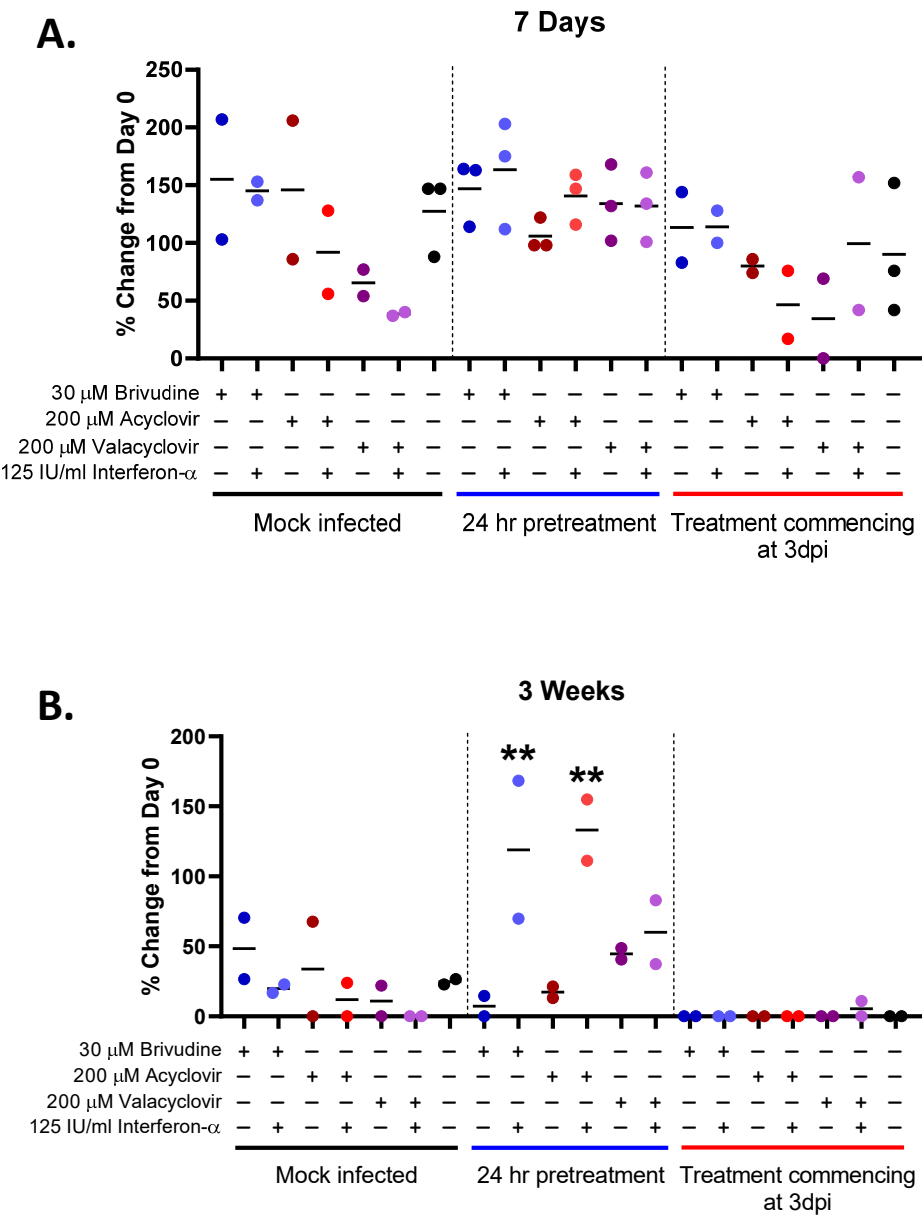

Supplement: S6 Fig — Viability of organoids (Prestoblue metabolism assay) after infecting with 0.00001 MOI McKrae HSV1 for (A) 7 days and (B) three weeks with the indicated antiviral treatments either started 24 hrs before infection and continued for 15 days (to 14dpi) or started at 3 dpi and continued to 14 dpi. Dots show individual organoids and means are indicated by bars. One way ANOVA analysis with Tukey’s secondary testing, comparing viability of the treatments with the untreated infected control, showed no significant changes in viability at 7 days (A) and at 3 weeks (B) only the Brivudine and Acyclovir treatments co-administered with interferon-α 24 hrs prior to infection remained significantly viable. (PDF) [file pone.0277051.s006.pdf]

S7 Fig

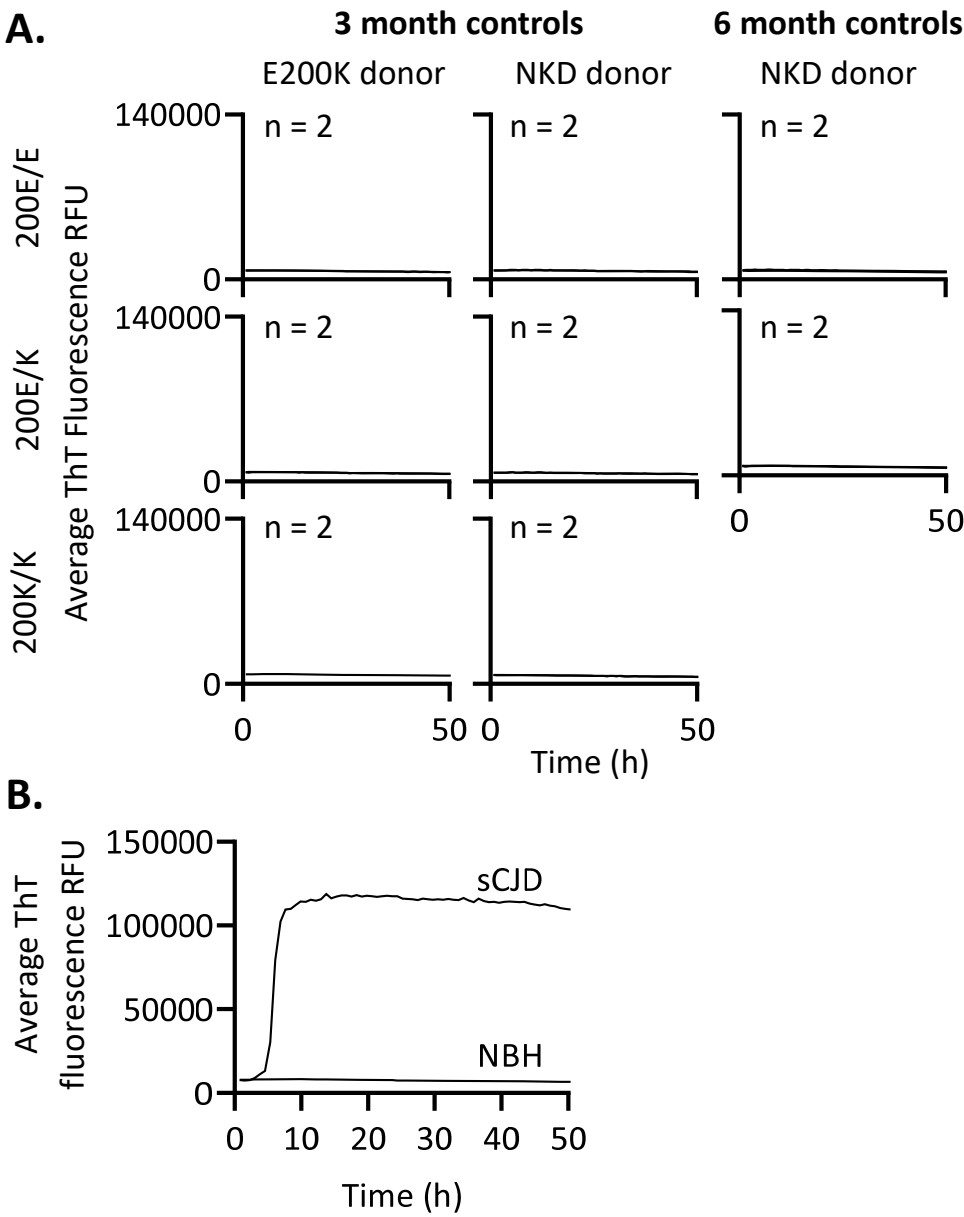

Supplement: S7 Fig — Untreated control organoids were collected at the same timepoints as the test organoids for RT-QuIC analysis, and sCJD infected organoids were included as a positive control. A. Control isogenically matched organoids collected at 3 and 6 months of latent HSV1 infection. Traces shown are averages of 4 replicate reactions per ‘n’ individual organoids, with the ‘n’ indicated on each graph. B. Example sCJD (positive) and normal brain homogenate (NBH; negative) infected organoid control reactions. (PDF) [file pone.0277051.s007.pdf]
