# Supplementary material for: Stress and viral insults do not trigger E200K PrP conversion in human cerebral organoids
Source: PLoS One. 2022 Oct 27;17(10):e0277051. doi: 10.1371/journal.pone.0277051 (PMC9612459; doi:10.1371/journal.pone.0277051)
Supplement: S1 Raw images — (PDF) [file pone.0277051.s008.pdf]

Panel shown in Figure 2A

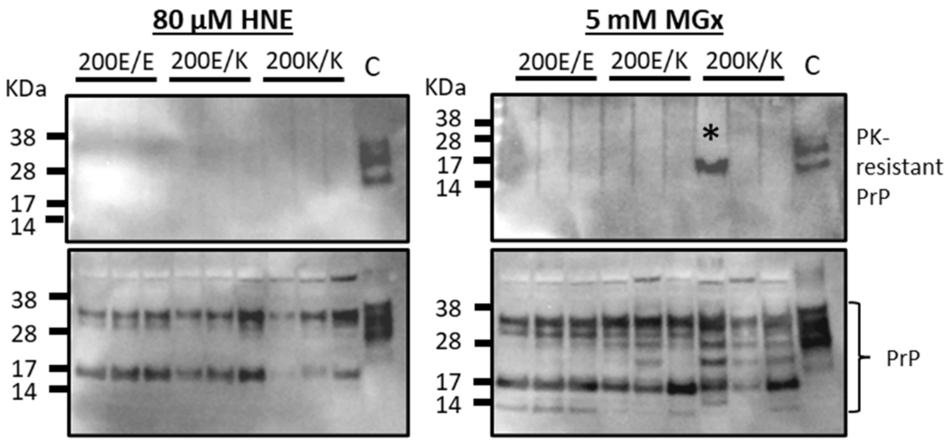

Raw blot images spatially corresponding with cropped blots above

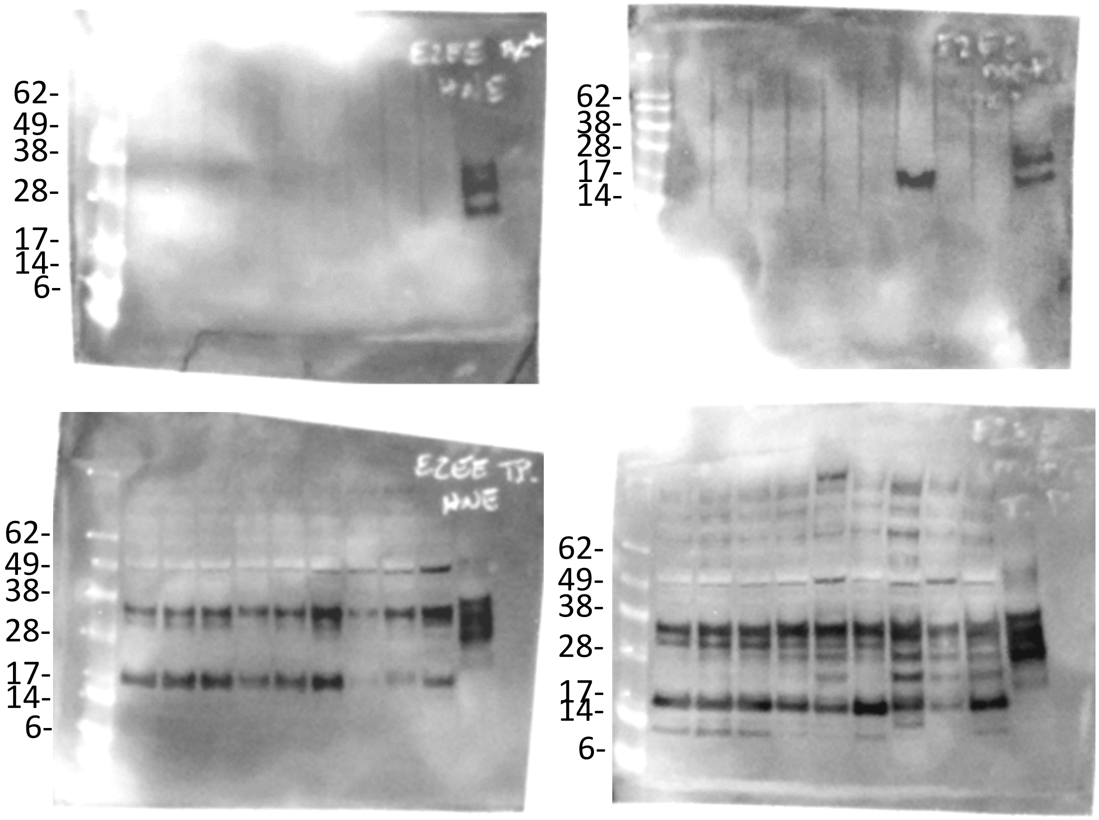

Ladder = see blue plus 2 (Invitrogen)

Panel shown in Figure 3B

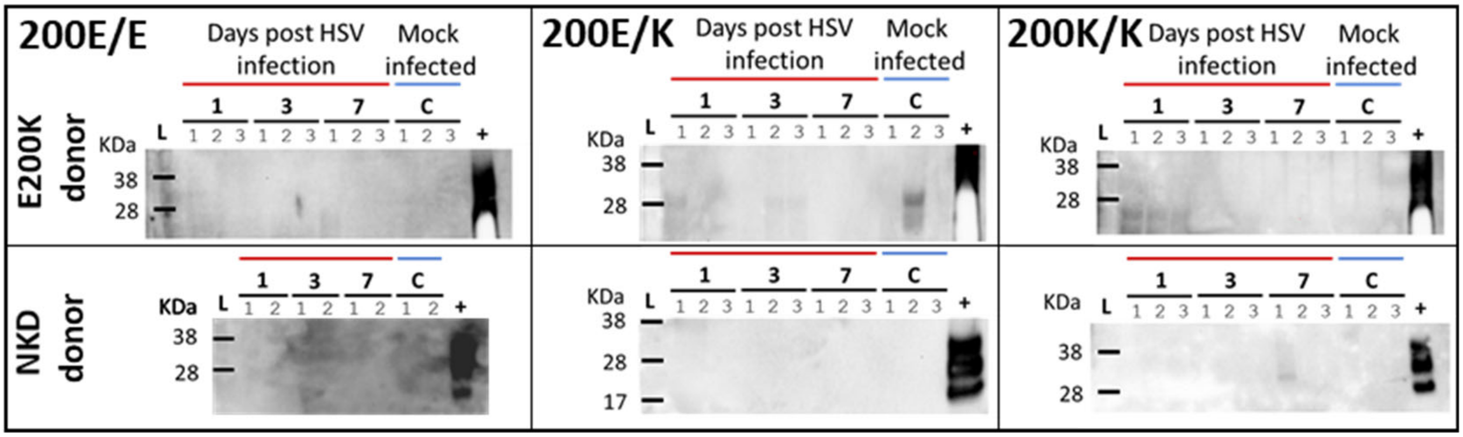

Raw blot images spatially corresponding with cropped blots above

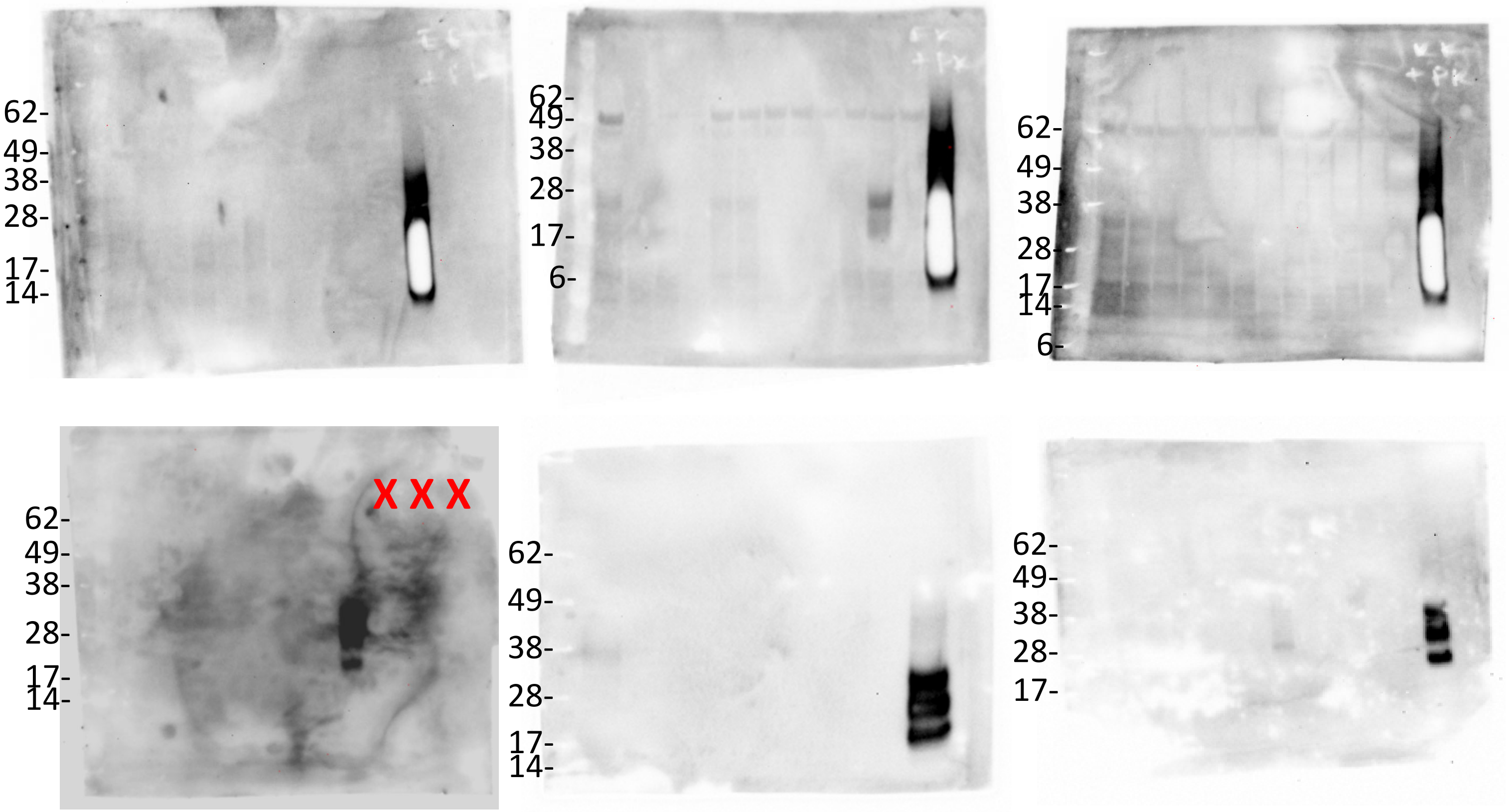

X = lane not used in current manuscript. Ladder = see blue plus 2 (Invitrogen)

Panels shown in Figure 4 B & E

Raw blot images spatially corresponding with cropped blots above

3 months

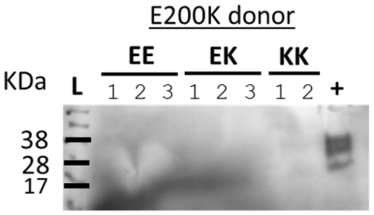

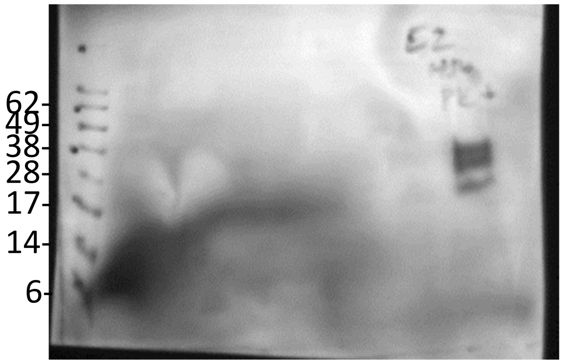

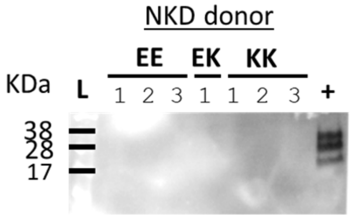

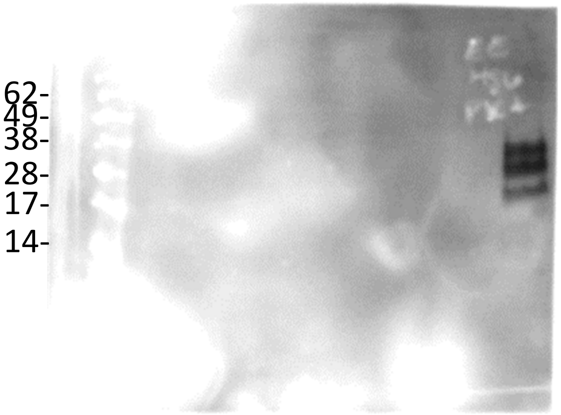

6 months

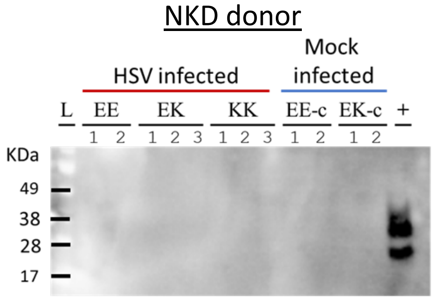

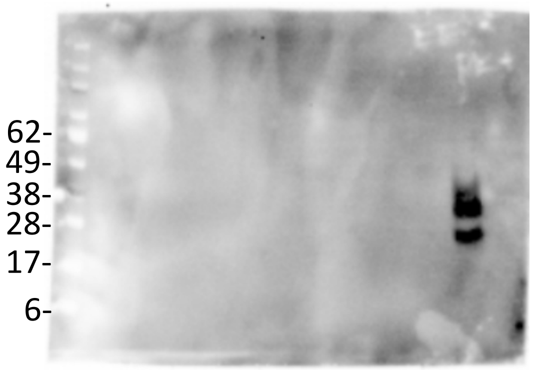

Ladder = see blue plus 2 (Invitrogen)
